# ‘Worried to death’: the assessment and management of anxiety in patients with advanced life-limiting disease, a national survey of palliative medicine physicians

**DOI:** 10.1186/s12904-017-0245-5

**Published:** 2017-12-11

**Authors:** N. Atkin, V. Vickerstaff, B. Candy

**Affiliations:** 1 Camden, Islington ELiPSe and UCLH & HCA Palliative Care Service, London, UK; 2 Marie Curie Palliative Care Research Department (MCPCRD), Division of Psychiatry, University College London, 6th Floor Wing B, Maple House, 149 Tottenham Court Road, London, W1T 7NF UK

**Keywords:** Palliative care, Anxiety, Health care surveys, Physicians

## Abstract

**Background:**

Anxiety adversely affects quality of life and is common in adults with advanced life-limiting disease. There are no UK-wide guidelines on the assessment and management of anxiety in this specific population and there is little evidence regarding drug treatments. This study aimed to explore how palliative care physicians assess and manage anxiety in their patients, and to identify barriers encountered.

**Methods:**

A cross-sectional survey was undertaken of all physicians working in specialist palliative care in the UK who were members of the Association for Palliative Medicine. This was conducted in February 2014 using an online questionnaire.

**Results:**

The response rate was 23% (230/980) and 61% of respondents were consultants. Most did not use tools to screen for anxiety (87%) and almost all used the clinical interview to diagnose anxiety (99%). Only 8% used psychiatric criteria. Most physicians reported difficulties managing anxiety (93%). Only 33% thought they had adequate training in this area. Most had difficulty accessing psychological and/or psychiatric services (71%, 64% respectively). The majority used a combination of pharmacological and non-pharmacological treatments for anxiety. The most frequently prescribed first-line medications for patients with a prognosis of days to weeks were benzodiazepines (93%), usually lorazepam. The use of benzodiazepines over antidepressants was statistically significant (*p* < 0.001). For patients with a prognosis of months, antidepressants were most frequently prescribed first-line (60%), significantly more than benzodiazepines (*p* < 0.001). However, benzodiazepine use was still common in this prognostic group with 47% prescribing it first-line, sometimes in combination with an antidepressant.

**Conclusion:**

This is the first national survey on the assessment and management of anxiety in palliative care. Findings demonstrate the infrequent use of screening tools, variation in prescribing practice, potentially inappropriate use of benzodiazepines for patients with a prognosis of months, training gaps and poor access to psychological and psychiatric services in the UK. This highlights the need for formal training, further research into the pharmacological management of anxiety in this population and evidence-based national guidance to support clinical decision-making and service development.

## Background

Anxiety is common in adults with advanced life-limiting disease, adversely affecting quality of life, social relationships and daily functioning at a critical time [1, 2]. It impairs the individual’s ability to cope with their illness, erodes their trust in physicians, reduces treatment compliance and makes physical symptoms more difficult to manage [1–3]. Estimates of the prevalence of anxiety disorder in the palliative care setting range from 6.8 to 13.3% [4], while significant anxiety symptoms are more common (24–48% [5–7]). However, anxiety is frequently unrecognised and untreated [1, 8, 9].

Anxiety is a future-oriented mood state associated with preparation for possible, upcoming negative events [10]. Pathological anxiety is persistent or recurrent anxiety, fear or worry in excess of what is to be expected in the individual’s situation and causing clinically significant distress or impaired functioning [11–13]. Current psychiatric classifications recognise anxiety as a group of disorders including generalised anxiety disorder (GAD), panic disorder, phobias, specified and unspecified anxiety disorder [13, 14]. Anxiety symptoms may also be the prominent feature in stressor-related disorders such as adjustment disorder [13]. However, it can be difficult to apply the strict criteria for specific anxiety disorders to patients with advanced life-limiting disease, although they experience symptoms of anxiety that cause significant distress or functional impairment [12]. A broad definition of anxiety, incorporating all these anxiety states, is most relevant and practical in the palliative care clinical setting.

A range of screening tools have been developed to help physicians identify patients with significant anxiety who need a more thorough diagnostic interview. The most extensively validated screening tool in palliative care populations is the Hospital Anxiety and Depression Scale (HADS) [15–17]. There are recent guidelines for cancer patients that have recommended other screening tools such as the Edmonton Symptom Assessment System (ESAS), ESAS Revised (ESASr), Generalised Anxiety Disorder 7-item Scale (GAD-7) and the Distress Thermometer (DT) [18–20]. Structured diagnostic interview tools have also been developed, such as the Structured Clinical Interview for the Diagnostic and Statistical Manual of Mental Disorders Fifth Edition (SCID-5), but the validity of diagnostic tools in the palliative care setting is questionable [21].

There are no national or international guidelines for the assessment and management of anxiety in palliative care patients. Considering the guidance for related populations, there are Canadian, American and Australian evidence-based guidelines on the assessment and management of anxiety and depression in adult cancer patients [18–20, 22]. These vary in detail and specificity but all recommend routine screening for anxiety at regular intervals with validated tools. They advise an individually tailored, stepped care model of intervention, frequently involving a combination of psychological and pharmacological treatments for moderate to severe anxiety symptoms. The Australian guidelines provide the most specific recommendations regarding medication, advising selective serotonin reuptake inhibitors (SSRIs) as first-line pharmacotherapy and advising against benzodiazepine use due to risk of dependence, tolerance and other morbidities including confusion, ataxia, falls in the elderly and rebound anxiety [20]. However, they advise benzodiazepines can be used in crisis situations, including palliative care. The National Institute for Health and Care Excellence (NICE) Guidance on Improving Supportive and Palliative Care for Adults with Cancer provides UK-based guidance on psychological distress management in cancer patients [9]. While not addressing anxiety specifically, it recommends that cancer patients experiencing significant psychological distress be referred to specialist psychological support services. It proposes a four-level model of psychological assessment and intervention but does not provide specific guidance on pharmacological management.

While guidance regarding the assessment and management of anxiety specifically in the palliative care population is lacking, so too is high-level evidence in this area. Our Cochrane review, recently updated, and Nübling et al.’s systematic review both conclude that there is inadequate evidence to make any recommendations for pharmacological treatment of anxiety in the palliative care population [23–25]. Considering the evidence base in other populations, research into anxiety treatments within the cancer population has predominantly been undertaken in patients with early stage cancer [26]. There is randomised controlled trial (RCT) evidence for benzodiazepine and antidepressant efficacy in the heterogeneous cancer population [26, 27]. These RCTs demonstrate short-term benzodiazepine efficacy (10 days) but no difference compared to placebo at 4 weeks. For the management of GAD in the general adult population, RCTs demonstrate the efficacy of antidepressants, benzodiazepines and other medications, but the evidence base for benzodiazepines is much smaller than for antidepressants and there is an increased risk of discontinuation for lorazepam, due to side effects, as well as evidence of dependence [28]. Therefore NICE recommends that benzodiazepines are not prescribed for GAD in the general adult population in the UK, except for short-term use for 2–4 weeks [28]. However, given the differing needs, physical fitness and prognosis of palliative care patients compared to the general population or heterogeneous cancer population, it is difficult to know how much can be extrapolated from the existing data and applied to the palliative care population.

While there is a paucity of high-quality evidence for pharmacological treatments for anxiety in the palliative care population, non-pharmacological therapies have been studied in this specific population. This research suggests that anxiety improves with cognitive behavioural therapy (CBT) [29, 30], group support [31], counselling [32] and some complementary and alternative therapies [33–35]. There is little research directly comparing pharmacological and non-pharmacological treatment for anxiety in palliative care [27]. As in the general population, a combination of pharmacological and psychological therapies may be more effective than either treatment alone [36, 37], but the data to support this is limited.

In the absence of a solid evidence base or guidelines specific to the treatment of anxiety in patients with advanced life-limiting disease, this study aims to establish, through a UK-wide survey, how palliative medicine physicians currently assess and manage anxiety in their patients. In doing so, it aims to provide vital information needed to develop national guidelines, improve training and services and plan relevant research in this area.

## Methods

### Study design

A cross-sectional survey design using an online platform to collect quantitative data and some qualitative data.

### Survey development

A literature review and previous surveys [38, 39] informed the development of a twenty-eight item web-based questionnaire. The questionnaire was reviewed by experts in palliative medicine and psychiatry, and pilot-tested on seven palliative medicine physicians of varying grades. Questions were revised accordingly.

For the purpose of the survey, anxiety was defined as persistent or recurrent anxiety causing clinically significant distress or functional impairment. This included anxiety and stressor-related disorders defined by psychiatric criteria, as well as other significant anxiety states.

The questionnaire consisted of 6 sections:

1. Informed consent
2. Demographic details of participants; work setting, years of palliative care experience and previous psychological/psychiatric training.
3. Process by which participants diagnosed anxiety in their patients.
4. Methods participants used to manage anxiety, including pharmacological and non-pharmacological treatments in patients with shorter and longer prognoses.
5. Challenges encountered when assessing and managing anxiety.
6. Access to psychological, psychiatric and other support services.

Twenty-three questions were multiple choice with options for additional free text comments. Five were open questions with free text responses. No personal data was collected.

### Sampling

The sample cohort (*n* = 980) comprised of all physicians working in adult specialist palliative care in the UK, who were members of their specialist society (The Association for Palliative Medicine of Great Britain and Ireland, APM). They were identified via the APM database. The cohort included physicians working in all specialist palliative care settings: inpatient palliative care unit, hospital consult service, community service, outpatient service and day unit. All grades of physician were included: consultants, registrars (training in specialist palliative care) and non-training career grade physicians (known as specialty staff grade and associate specialists, SSAS). Retired APM members and those working outside the UK were excluded.

### Survey administration

In order to ensure anonymity, the APM directly emailed the survey web link to each physician in February 2014. To increase response rate, a reminder email was sent four weeks later and the survey was open for five weeks. The survey was administered online via SurveyMonkey, an online survey development company. The survey could only be completed once by each participant to avoid duplicate surveys.

### Data analysis

Only completed surveys were included in the analysis. To ensure a representative sample of UK specialist palliative medicine physicians, we compared respondents’ specialty grades to those of the total eligible APM membership and the total UK specialist palliative medicine workforce, as estimated by the Royal College of Physicians census [40].

Using Stata Version 12 [41] we described the quantitative responses using percentages. Percentages were based on the number of respondents answering each question and were rounded to the nearest whole number. Pearson Chi², or Fisher’s exact test where appropriate, were used to compare groups. Multiple tests were carried out without adjustments and a *p* value of < 0.05 was used as the level of significance. Free text responses were explored by one researcher (NA) using thematic content analysis, and themes generated were verified by a second researcher (BC).

## Results

Of 980 surveys emailed, a total of 279 respondents commenced the survey, 231 completed it and one of these was excluded as they did not meet the eligibility criteria. Overall, the response rate was 23% (230/980). The majority of respondents were consultants (61%) and the most common work setting was the inpatient palliative care unit (54%). Further respondent characteristics are reported in Table 1.

**Table 1:** Characteristics of respondents

Characteristics		*n* (%) (*n* = 230)
Grade	Consultant	141 (61)
	SSAS	48 (21)
	Registrar	41 (18)
Years palliative care experience	<5	41 (18)
	5–10	61 (27)
	11–20	102 (44)
	>20	26 (11)
Main work setting	Inpatient palliative care unit	124 (54)
	Hospital consult service	48 (21)
	Community	27 (12)
	Outpatient/day unit	6 (3)
	Equal split across settings	25 (11)
Geographical location	England	188 (82)
	Scotland	20 (9)
	Wales	15 (7)
	Northern Ireland	5 (2)

*Abbreviations: SSAS* specialty staff grade and associate specialists

Comparison by staff grade with the total APM sample cohort showed that a slightly higher proportion of respondents were consultant or SSAS grade, rather than specialty registrar grade. Of the APM’s membership (*n* = 980) at the time of the survey, 30% (141/472) of consultants responded, 33% (48/146) of SSAS physicians responded and 17% (41/244) of registrars responded. However, 118 members registered with the APM were recorded as ‘unknown grade’ but those who completed the survey all documented a specific grade, and physicians may not have updated their APM records when progressing from one grade to another, introducing some inaccuracy into these percentages. Considering grade of respondents in relation to the estimated total UK specialist palliative care physician workforce [40], there was a similar over-representation of more senior physicians and under-representation of trainees. Twenty-eight percent (141/502) of consultants in the UK responded and 18% (41/222) of registrars in the UK responded. Total SSAS workforce data was not available. It was not possible to compare other variables due to missing data.

### Assessment of anxiety

Anxiety was common, with the majority of physicians diagnosing it in a quarter or more of their patients (Table 2). Only a proportion of physicians (28%) were aware of local or network guidelines. Most did not use screening tools for anxiety (87%) and used the clinical interview to diagnose anxiety (99%). A small minority used psychiatric criteria (8%) or a diagnostic tool (12%) in addition to the clinical interview for diagnosis. Where a tool was used, the HADS was the most popular for screening and diagnosis (52% and 81%, respectively).

**Table 2:** Assessment of Anxiety

		*n* (%) (*n* = 230)
% of patients with anxiety	<25	69 (30)
	25–50	109 (47)
	>50	34 (15)
	Unsure	18 (18)
Have local or network guideline		65 (28)
Use screening tool routinely		29 (13)
Screening tool used (*n* = 29)	HADS	15 (52)
	DT	7 (24)
	ESAS	2 (7)
	Other tool	5 (17)
Diagnose using (tick all that apply)	Clinical interview	227 (99)
	DSM-5 or ICD-10	18 (8)
	Diagnostic tool	27 (12)
Diagnostic tool used (*n* = 27)	HADS	22 (81)
	GAD-7	1 (4)
	BEDS	2 (7)
	Other tool	2 (7)
Routinely assess suicide risk when anxiety diagnosed		79 (34)

*Abbreviations: DSM-5* Diagnostic and Statistical Manual of Mental Disorders Fifth Edition, *DT* Distress Thermometer, *ESAS* Edmonton Symptom Assessment System, *GAD-7* Generalised Anxiety Disorder 7-item Scale, *HADS* Hospital Anxiety and Depression Scale, *ICD-10* International Statistical Classification of Diseases Tenth Revision, *BEDS* Brief Edinburgh Depression Scale

### Management of anxiety

Physicians were asked about their management strategies for two different groups of patients, those with a likely prognosis of days to weeks (d-w) and those with a likely prognosis of months (m).

All but one respondent reported that they treated anxiety when diagnosed. A small proportion recommended only non-pharmacological treatments first-line, usually for patients with a longer prognosis (4% d-w, 14% m). Also, a few respondents recommended only pharmacological treatments first-line, usually for patients with a shorter prognosis (8% d-w, 2% m). However, the majority used a combination of pharmacological and non-pharmacological treatments first-line for both groups (88% d-w, 84% m). The majority of physicians (56%) adopted the same management approach for patients with specific anxiety disorders defined by psychiatric criteria as they did for other patients with anxiety. Where management changed, this was most often in seeking psychiatric input.

### 1. Non-pharmacological Management

**Fig. 1:**
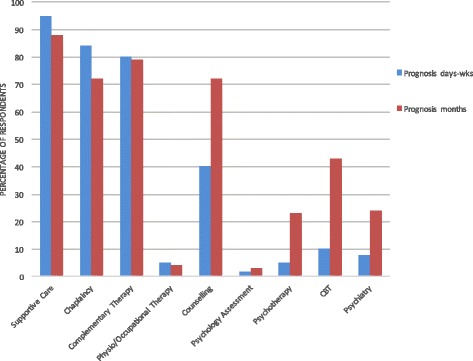
Non-pharmacological management of anxiety

When non-pharmacological management was offered, this usually involved a combination of multiple non-pharmacological therapies. Supportive care from the palliative care team, chaplaincy input and complementary therapies were recommended for most patients in both prognostic groups (Fig. 1). Psychological therapies and psychiatric input were more frequently recommended for those with a longer prognosis. Of these, counselling was most commonly offered (40% d-w, 72% m), followed by CBT (10% d-w, 43% m), psychiatry input (8% d-w, 24% m) and psychotherapy (5% d-w, 23% m).

### 2. Pharmacological Management

First and second-line medications for each group are reported in Table 3.

#### Prognosis days to weeks

Benzodiazepines were the most common first-line anxiolytic medication for patients with a prognosis of days to weeks (93%). Use of these in preference to antidepressants was statistically significant (*p* < 0.001).

Where a single benzodiazepine was specified, this was usually lorazepam (86%), followed by diazepam (7%) and midazolam (5%). The most common second-line drug choice was an alternative benzodiazepine (70%), most often midazolam.

Where medication dosing regimes were specified, lorazepam was usually prescribed ‘as needed’/pro re nata (PRN*)* rather than regularly (84% PRN, 16% regularly) and given via the sublingual route (72%). The most common starting dose was 0.5-1 mg (53%) but PRN doses ranged from 0.5 mg to 2 mg with a maximum frequency of twice daily to hourly. Midazolam doses were more variable, with PRN starting doses ranging from 1.25 mg to 10 mg and continuous infusion starting doses ranging from 2.5 mg to 15 mg over 24 h. Diazepam starting doses ranged from 2 mg to 10 mg, with frequencies of once to four times daily.

#### Prognosis months

The majority of respondents (63%) used a different drug regime for those whose prognosis was months. Antidepressants were the most common first-line anxiolytic medication (60%). Use of these in preference to benzodiazepines was statistically significant (*p* < 0.001) but use of benzodiazepines was still common (47%, some in combination with antidepressants). Of the antidepressants, citalopram was most frequently prescribed (54%), followed by mirtazapine (38%) and sertraline (6%). The most common second-line medication choice was a benzodiazepine (46%), usually lorazepam (59%).

For both prognostic groups other drugs used first-line included haloperidol, levomepromazine, promazine, pregabalin, oxazepam, duloxetine and nortriptyline. There was even greater variation in second-line drugs.

**Table 3:** Pharmacological Management of Anxiety

Most frequently prescribed medication		Prognosis days-weeks	Prognosis months
		*n* (%)	*n* (%)
**1st Line**		**(** ***n*** **= 216)**	**(** ***n*** **= 211** **)**
	**Benzodiazepine (BZ)**	**200 (93)**	**99 (47)**
	Alone	188 (87)	78 (37)
	Combined (with another drug class)	12 (6)	21 (10)
	Where BZ specified:	(*n = 147*)	(*n = 73*)
	Lorazepam	127 (86)	60 (8)
	Diazepam	11 (7)	13 (18)
	Midazolam	8 (5)	0 (0)
	**Antidepressant (AD)**	**21 (10)**	**127 (60)**
	Alone	12 (6)	107 (51)
	Combined (with BZ)	9 (4)	20 (9)
	Where AD specified:	(*n* = 17)	(*n* = 93)
	Mirtazapine	12 (71)	35 (38)
	Citalopram	3 (18)	50 (54)
	Sertraline	2 (12)	6 (6)
	**Other Medications**		
	Haloperidol	4 (2)	2 (1)
	Levomepromazine	2 (1)	0 (0)
	Pregabalin	0 (0)	4 (2)
**2nd Line**		**(** ***n*** **= 142)**	**(** ***n*** **= 124)**
	**Benzodiazepine**	**99 (70)**	**57 (46)**
	Where BZ specified:	(*n* = 96)	(*n* = 49)
	Midazolam	46 (48)	4 (8)
	Diazepam	31 (32)	16 (33)
	Lorazepam	18 (19)	29 (59)
	**Antidepressant**	**26 (18)**	**55 (44)**
	Where AD specified:	(*n* = 18)	(*n* = 41)
	Mirtazapine	9 (50)	22 (54)
	Amitriptyline	4 (22)	1 (2)
	Citalopram	3 (17)	11 (27)
	Sertraline	1 (6)	5 (12)
	**Other Medications**		
	Levomepromazine	9 (6)	3 (2)
	Pregabalin	2 (1)	8 (6)
	Morphine	2 (1)	3 (2)
	Unspecified antipsychotic	3 (2)	0 (0)

Medications used by *n* = 1 or less respondents in both prognostic groups not included in table

*n* numbers: variation as non-compulsory free text answers so some respondents answered only part of question, some specified drug and others only gave drug class

### Challenges encountered and access to support services

**Fig. 2:**
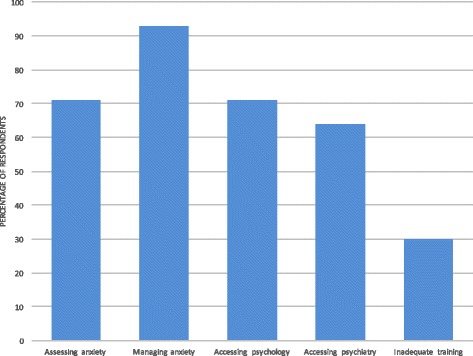
Difficulties encountered by respondents

The majority of physicians reported difficulties assessing anxiety (71%; Fig. 2), particularly differentiating a pathological state from a ‘normal’ reaction to diagnosis or impending death. Almost all physicians found anxiety difficult to manage (93%). The most commonly reported challenges were accessing timely non-pharmacological therapies, managing medication side-effects and refractory anxiety. Thirty percent thought they did not have adequate training in the assessment and management of anxiety, and a further 37% were unsure whether they did. Those with more years of experience in palliative care or a more senior grade were more likely to feel adequately trained (*p* = 0.04, *p* = 0.02). Previous psychiatric or psychological training and a more senior grade were associated with less difficulty assessing anxiety (*p* = 0.04, *p* = 0.001). However, as almost all respondents reported difficulties managing anxiety, there was only a trend towards reduced difficulties with previous training, years of experience and more senior grade (*p* = 0.57, *p* = 0.16, *p* = 0.07).

Most physicians had direct access to chaplaincy, complementary therapies and counselling (96, 90, 73% respectively). However, direct access to psychology and psychiatry was less common (45, 44%) and the majority of physicians reported difficulties accessing these services (71, 64%). In the case of psychology and CBT services, difficulties were most often due to inappropriately long waiting times for appointments for patients with a short prognosis, no local service provision or staff shortages. The main challenges regarding access to psychiatric services were the services’ reluctance to see palliative care patients, particularly in the hospice setting, inappropriately long waiting times to be seen and the lack of formal or direct referral systems.

Many respondents reported initiatives within their own services that had improved their clinical management of anxiety, or suggested changes they felt would address current challenges. These included additional training for themselves (formal teaching, CBT training, psychiatric placements), having in-house staff CBT trained, employing a psychologist or psychiatrist part-time to review patients and upskill the team, developing relationships and service level agreements with local psychological and psychiatric services, and the creation of national guidelines.

## Discussion

This survey demonstrates that the majority of palliative care physicians find anxiety difficult to manage, although it is so common in their patient population. The findings highlight several key issues, which may be amenable to improvement, including the infrequent use of validated screening tools, wide variation in prescribing practice, potentially inappropriate use of benzodiazepines, gaps in training and poor access to mental health services.

Our results show that most palliative care physicians in the UK are not using standardised, validated tools to assess anxiety. Although there is a paucity of data on the impact of screening for anxiety in the palliative care population, there is RCT evidence demonstrating the benefits of screening in the cancer population, if appropriate follow-up can be provided [42]. Recent evidence-based guidelines for cancer patients all recommend routine screening for anxiety using validated tools, followed by clinical interview when screening positive [18–20]. While the palliative care population differs from cancer population, whose disease is at various stages, this evidence and international consensus for the cancer population raises the possibility that screening tools should be used in the palliative care population too, particularly as anxiety is under-diagnosed [1, 8, 9]. Meanwhile, evidence for the validity of diagnostic (case-finding) tools is lacking in both palliative care and cancer populations [21, 43]. Despite this, our study shows that some palliative care physicians in the UK are using tools such as the HADS for diagnosis. These findings confirm the need for further study of assessment tools for anxiety in the palliative care population and for specific guidelines to support palliative medicine physicians in the UK.

Our study demonstrates widespread use of benzodiazepines for anxiety management, even for palliative care patients with a prognosis of months. Although there are no national guidelines specific to this population, the 2014 NICE Quality Standard for Anxiety Disorders and American and Australian guidelines for cancer patients all advise against prescribing benzodiazepines for anxiety except in short-term crisis situations, due to significant adverse effects and the development of tolerance and dependence [19, 20, 44]. While the evidence base for the pharmacological management of anxiety in the palliative care population is lacking, data from cancer populations demonstrates the short-term (days to weeks) rather than longer-term benefit of benzodiazepines [27]. In the general adult population the evidence base for benzodiazepines is smaller than that for anxiolytic antidepressants and the risk of side effects greater [28]. In view of this significant body of research and the overlap between the heterogeneous cancer population and palliative care patients with a prognosis of months, it is possible that the common use of benzodiazepines in this latter group, as identified in our study, may not be the safest or most effective treatment approach. Our results also demonstrate substantial variation in drug choice and dosing, a further indication that some patients may not be receiving the most appropriate treatment. These issues highlight a major gap in the evidence base for anxiety management in palliative care and the need for UK guidance on prescribing for these patients.

Our results show that despite anxiety being a common problem among palliative care patients, almost all palliative care physicians, even the most experienced, have difficulty managing anxiety. As the majority of physicians feel they do not have adequate training in this area, or are unsure whether they do, our findings suggest gaps in training may be contributing to their difficulties and potentially impacting on patient care. Survey respondents identified that further training, closer links with experts and relevant guidelines would be beneficial, which is consistent with other studies of physician-perceived barriers to psychosocial care at the end of life [45, 46].

However, the most common difficulty respondents reported with regards to anxiety management was lack of access to timely non-pharmacological therapies, particularly from psychology and psychiatry services. This is contrary to NICE guidance which recommends that cancer patients (irrespective of disease stage) experiencing significant psychological distress should be offered prompt referral to specialist psychological support services [9]. It also recommends that all palliative care teams should have access to these services, close working relationships with mental health teams and adequate training in providing general psychological support. Price et al.’s survey of the availability of psychological services in hospices in the UK and Ireland in 2005 found that there was inadequate access to psychologists and psychiatrists [47]. Our survey, conducted 9 years later, demonstrates some improvement in service provision. Physicians working in hospices now have dramatically improved access to counselling for their patients (76% in 2014 versus 38% in 2005). However, despite the NICE guidance, there has been only a marginal improvement in access to psychologists and psychiatrists in hospices over this period (46% versus 41% for psychology, 37% versus 30% for psychiatry).

The issues identified in our survey are not unique to the UK or the hospice setting. Patterson et al.’s 2014 survey of American hospital-based palliative care consult services demonstrated that most respondents (71%) would like psychiatry to be more involved with the palliative care service than they currently are, and the barriers identified were similar to our study [48]. However, the American survey found that 72% of consult services had some involvement with a psychiatrist, suggesting better engagement between psychiatry and palliative care than in the UK, where only 56% of the hospital-based palliative care physicians in our survey had direct access to psychiatry. In support of improved psychiatric involvement, Patterson’s survey demonstrated that palliative care teams with an identified psychiatrist were more likely to report that their patients’ mental health needs were being met.

### Limitations

The major limitation of this study was the low response rate, despite reminder emails to all physicians. This is unfortunately common for web-based surveys distributed by email [49]. It is challenging to access these professionals at a national level as they work in a range of different organisations and settings. However, as the first survey on this topic, a national web-based survey method was chosen to give the broadest reflection of UK-wide practice and act as a starting point for more detailed, targeted study. We are aware that this low response rate may impact on the representativeness of the sample and increases the risk of response bias. The results are more likely to reflect the views of physicians with an interest in anxiety and it is therefore possible that the knowledge gaps and training needs are even greater than those identified in the study. Representativeness of the sample is further affected by the disproportionately higher response rate from consultants and SSAS physicians than registrars, more junior physicians. This may have impacted on our estimate of the knowledge gaps and training needs of the UK specialist palliative care physician workforce. However, accurate analysis of the representativeness of the sample in this study is limited by missing UK workforce and APM membership data.

There were several other limitations. Due to the survey format, this study relied on respondents’ subjective reports of their practice, rather than more objective and prospective assessments. The use of some survey questions with free text answers meant those responses were open to interpretation when categorising and analysing the data. Moreover, some optional, clarifying comments were made by only a small proportion of respondents so it is difficult to draw valid conclusions from those qualitative responses. Also, as part of the statistical analysis multiple tests were carried out without adjustments as it was an exploratory analysis. Finally, the survey was conducted in 2014 and it is possible that there have been changes in practice since, particularly as new or updated guidelines (for cancer patients and the general population) were produced shortly before and after the data collection [18–20, 24].

## Conclusion

To our knowledge this is the first national survey exploring how palliative medicine physicians assess and manage anxiety in their patients. It highlights the infrequent use of screening tools, substantial variation in prescribing practice, potentially inappropriate use of benzodiazepines in patients with a prognosis of months, training gaps and poor access to psychological and psychiatric services in the UK. This suggests that palliative medicine physicians should receive formal training in the management of anxiety and develop local referral networks with mental health services. The findings also highlight the urgent need for further research into the pharmacological management of anxiety in the palliative care population. The development of a UK-wide guideline is an essential next step in supporting clinical decision-making, service development and ultimately improving patient care.

## Abbreviations

AD: Antidepressant
APM: Association for Palliative Medicine of Great Britain and Ireland
BEDS: Brief Edinburgh Depression Scale
BZ: Benzodiazepine
CBT: Cognitive behavioural therapy
DSM-5: Diagnostic and Statistical Manual of Mental Disorders, Fifth Edition
DT: Distress Thermometer
d-w: Days to weeks
ESAS: Edmonton Symptom Assessment Scale
ESASr: Edmonton Symptom Assessment Scale revised
GAD: Generalised anxiety disorder
GAD-7: Generalised Anxiety Disorder 7-item Scale
HADS: Hospital Anxiety and Depression Scale
ICD-10: International Statistical Classification of Diseases Tenth Revision
m: Months
NICE: National Institute for Health and Care Excellence
PRN: Pro re nata
RCT: Randomised controlled trial
SCID-5: Structured Clinical Interview for the Diagnostic and Statistical Manual of Mental Disorders Fifth Edition
SSAS: Specialty staff grade and associate specialists
SSRI: Selective serotonin reuptake inhibitor
